# Functional connectivity in burnout syndrome: a resting-state EEG study

**DOI:** 10.3389/fnhum.2025.1481760

**Published:** 2025-02-03

**Authors:** Natalia Afek, Dmytro Harmatiuk, Magda Gawłowska, João Miguel Alves Ferreira, Krystyna Golonka, Sergii Tukaiev, Anton Popov, Tadeusz Marek

**Affiliations:** ^1^Doctoral School in the Social Sciences, Jagiellonian University, Kraków, Poland; ^2^Department of Electronic Engineering, Igor Sikorsky Kyiv Polytechnic Institute, Kyiv, Ukraine; ^3^Institute of Applied Psychology, Jagiellonian University, Kraków, Poland; ^4^Faculty of Medicine, University of Coimbra, Coimbra, Portugal; ^5^Institute of Public Health, Università della Svizzera italiana, Lugano, Switzerland; ^6^Educational Scientific Institute of High Technologies, Taras Shevchenko National University of Kyiv, Kyiv, Ukraine; ^7^Faculty of Applied Sciences, Ukrainian Catholic University, Lviv, Ukraine; ^8^Faculty of Psychology, SWPS University, Katowice, Poland

**Keywords:** functional connectivity, EEG, burnout syndrome, stress, resting state

## Abstract

Chronic occupational stress is associated with a pronounced decline in emotional and cognitive functioning. Studies on neural mechanisms indicate significant changes in brain activity and changed patterns of event-related potentials in burnout subjects. This study presents an analysis of brain functional connectivity in a resting state, thus providing a deeper understanding of the mechanisms accompanying burnout syndrome. The sample consists of 49 burnout employees and 49 controls, matched by age, gender and occupation (M_age_ = 36.15, SD = 8.10; 59 women, 39 men). Continuous dense-array EEG data were collected from a 256-channel EEG system. The difference in functional connectivity between burnout and control subjects was tested in the eyes-closed (EC) and eyes-open (EO) conditions using the resting-state paradigm. The results indicate significant differences in brain activity between the burnout and the control groups. The resting-state network of the burnout group is characterized by decreased functional connectivity in frontal and midline areas in the alpha3 sub-band (11–13 Hz) in an eyes-open condition. The most significant effect of decreased connectivity was observed in the right frontal brain area. For the first time, these analyses point to distinctive aspects of functional connectivity within the alpha3 sub-band in burnout syndrome. These findings provide insights into the neurobiological underpinnings of burnout syndrome and its associations with changed resting-state networks. The data on neural characteristics in burnout subjects may help to understand the mechanisms of decline in cognitive function and emotion regulation and to search for adequate methods of treatment.

## 1 Introduction

Burnout syndrome is defined as a prolonged psychological reaction to chronic work-related stress (Maslach et al., 2001). In the 11th revision of the International Classification of Diseases (ICD-11), the World Health Organization (2019) classifies occupational burnout as a factor that influences health (code: QD85) and describes burnout syndrome as physical and mental exhaustion. Consistent with previous studies (Maslach and Jackson, 1981; Maslach et al., 1996), in ICD-11 burnout is characterized by three dimensions: (1) feelings of energy depletion; (2) increased mental distance from one’s job or feelings of negativism or cynicism related to one’s job, and (3) a sense of ineffectiveness and lack of accomplishment. Although burnout syndrome is still not recognized in ICD-11 as a disorder, in 14 European countries it has been acknowledged as an occupational disease (Canu et al., 2019; Lastovkova et al., 2018). In some countries, such as Sweden, it is recognized as a legal basis for sick leave (Friberg, 2009). According to the 6th European Working Conditions Survey, burnout is reported by 10% of European workers and 17% of workers in other countries included in the research (Schaufeli, 2018). Due to the prevalence of burnout and its economic and social costs (e.g., Blackburn et al., 2023), it is particularly important to understand the nature of this syndrome and conduct research on the underlying brain mechanisms.

Burnout has been studied as a separate syndrome for more than half a century, but the diagnosis is still not clear. In some studies, overlapping effects with depression and anxiety disorders are emphasized (e.g., Golonka et al., 2019b; Luijtelaar et al., 2010). According to Heinemann and Heinemann (2017), the reason for this ambiguity is the inconsistency and insufficient number of studies attempting to detect biomarkers of burnout as objective criteria. Establishing burnout-specific changes and possible abnormalities in brain activity is necessary to understand the phenomenon of burnout and may further enable it to be distinguished from other stress disorders, depression, alexithymia, or chronic fatigue syndrome. Using neuroimaging methodology and new approaches in data analysis, it is possible to extend the current knowledge of the mechanisms that underlie or are correlated with burnout syndrome, thus leading to a better understanding of this phenomenon and validation of the studied concept.

Functional magnetic resonance imaging (fMRI) studies have associated the severity of burnout symptoms with altered brain activation and brain structures. For example, in emotional exhaustion Tei et al. (2014) noticed decreased activation of the structures involved in empathic behavior [the anterior insula and inferior frontal gyrus cluster (AI/IFG) and the temporoparietal junction (TPJ)], while Durning et al. (2013) observed increased activity of the right posterior cingulate cortex and the middle frontal gyrus. Blix et al. (2013) revealed that the level of chronic occupational stress is inversely correlated with the volume of two structures of the striatum: the caudate nucleus and the putamen. Similarly, Gavelin et al. (2020) observed a significant decrease in caudate and putamen volumes in stress-related mental fatigue; Savic et al. (2018) found reduced caudate volumes (accompanying with enlarged amygdala volumes and thinning of the left superior temporal gyrus) in chronic occupational stress. This may be of particular importance because dopamine produced in the striatum is an important part of the brain reward system, which affects emotion regulation and motivational behaviors (Arias-Carrión et al., 2010). Additionally, Blix et al. (2013) found a significant decrease in the volume of the anterior cingulate cortex (ACC) and the dorsolateral prefrontal cortex (dlPFC) in patients with burnout. De Andrade et al. (2016) observed that high level of burnout was accompanied by increased activation of dlPFC in attention tasks. There are other fMRI studies which indicate associations between burnout/chronic occupational stress and disfunctions of PFC. For example, Abe et al. (2022) observed decrease in gray matter volume in the bilateral ventromedial prefrontal cortex (vmPFC) with the development of emotional exhaustion, and in the left vmPFC in increased depersonalization. This tendency was also observed by Savic et al. (2018) who pointed that impairment of the ability to regulate negative emotions in chronic occupational stress is associated with reduced thickness of the PFC.

Further evidence of changed characteristics of brain mechanisms in burnout syndrome comes from EEG studies. One of the first studies was conducted by Luijtelaar et al. (2010). In their analysis of event-related potentials (ERPs) in the oddball task with high (a “target”) and low (a “background”) tones, they observed reduced amplitude of the P300 component in the burnout group as a weaker response to the “target.” Additionally, in the resting state (2 min in eyes-open and eyes-closed conditions) Luijtelaar et al. (2010) performed a frequency-specific EEG power analysis, in which a lower alpha peak frequency and reduced beta power in burnout subjects were observed. Tement et al. (2016) used a resting-state eyes-closed condition (3 min) and focused on searching the relationships between EEG alpha frequency, burnout and depression. In the regression analysis they revealed a significant associations between individual alpha frequency (IAF) and depression, and between alpha power and burnout, suggesting that burnout may be a separate clinical syndrome. Subsequently, several experimental procedures have revealed differences in ERP characteristics between burnout and control groups (Golonka et al., 2017a, 2017b, 2019a; Sokka et al., 2014, 2017). In addition, in frequency-specific EEG power analyses, significant differences in a resting-state condition were observed, with reduced alpha band power in burnout individuals (Golonka et al., 2019a). Similarly, Yakovenko et al.’s (2021) study observed that the exhaustion stage is characterized by a decrease in the beta band power in the anterior area, which the authors associated with dysfunction of the frontal regulatory systems.

Although many studies have investigated localized changes in brain structures or activity patterns associated with burnout, increasing attention is being paid to how different cortical networks interact in real time (Friston, 2011). This shift toward a network-based perspective is particularly relevant for a multifaceted syndrome like burnout, which involves cognitive, emotional, and motivational alterations that may not be fully captured by traditional, region-specific approaches. Analyzing functional connectivity in resting-state data provides a powerful vantage point for identifying disruptions in baseline neural communication. Unlike task-based paradigms that can introduce confounds related to performance or motivation, resting-state EEG captures spontaneous activity, allowing for the examination of intrinsic network dynamics that may be uniquely compromised in burnout.

Existing fMRI data also indicate changes in brain functional connectivity in burnout participants. For example, Sandström et al. (2011) showed that cognitive impairments, such as decreased attention and worsened visuospatial memory, may be linked with dysfunction of the frontal/medial temporal cortex network as a result of stress-related exhaustion. In burnout individuals, the ability to downregulate negative emotions correlates with the weakening of functional connectivity between the amygdala and the anterior cingulate cortex, the dorsolateral prefrontal cortex, and the motor cortex (Golkar et al., 2014). Functional disintegration of the networks connecting the limbic system with the prefrontal cortex, as well as a decrease in the volume of the basal ganglia structures, has been revealed under clinical manifestations of exhaustion disorder (Grossi et al., 2015). According to Shang et al. (2022), burnout syndrome is associated with increased characteristic path length and decreased global efficiency, which suggests disrupted global integration of the functional network in burnout patients. However, not many EEG studies have explored brain connectivity in burnout syndrome.

Preliminary research on resting-state functional connectivity in emotional exhaustion among students was presented by Tukaiev et al. (2012, 2022), but only gender differences were reported; also, Harmatiuk et al. (2023) indicated significant differences in brain connectivity between exhausted students and controls in the alpha and gamma bands. Tement et al. (2016) explored similarities and differences between burnout and depression by analyzing coherence in the alpha band in burnout. They showed that burnout may be differentiated from depression by looking at alpha band (8–13 Hz) characteristics, i.e., individual alpha frequency and alpha power, but they did not observe differences in alpha sub-band connectivity when comparing burnout subjects and controls (Tement et al., 2016). All these studies, however, tested students only in an eyes-closed condition.

The aim of this study was to extend the existing knowledge on brain mechanisms in burnout syndrome by developing an analysis of functional connectivity in eyes-closed and eyes-open resting-state conditions for each EEG frequency band separately, as was previously done by Luijtelaar et al. (2010) and Golonka et al. (2019a) in spectral analyses. The eyes-open condition seems to be particularly promising because significant differences were found in this condition by both Luijtelaar et al. (2010) and Golonka et al. (2019a). The analysis of functional connectivity is based on coherence indices, which in most EEG studies refer to the square of the correlation coefficient between channels in specific frequency bands (Srinivasan, 1999). Coherence remains one of the most widely used and validated measures for identifying interdependencies among EEG signals, and it has proven effective in numerous clinical and research settings (Basharpoor et al., 2021; Bowyer, 2016; Fischer et al., 2023; Huang et al., 2023; Mano et al., 2022). Its simplicity in calculation and reproducibility enhances its suitability for burnout research. Moreover, coherence is particularly suited for analyzing synchronized oscillatory activity between distributed brain regions, offering valuable insights into potential disruptions in neural network communication associated with burnout (Bowyer, 2016). Additionally, the possibility of using a more accurate dense-array EEG system may make it possible to explore functional connectivity patterns in burnout more precisely, which is particularly significant when mapping functional connectivity due to possible artifacts (Lejko et al., 2020). Furthermore, this study will focus on a group of adult employees exhibiting burnout (as defined in ICD-11), characterized by three key symptoms: emotional exhaustion, cynicism/depersonalization, and inefficacy.

Employing EEG to investigate functional connectivity holds considerable potential as a meaningful step toward overcoming diagnostic obstacles we are facing in distinguishing burnout from other conditions. EEG recordings, with their high temporal resolution, enable researchers to capture rapid fluctuations in brain activity that may reflect disruptions in neural functioning associated with burnout syndrome. Specifically, functional connectivity measures can explain how burnout-related alterations influence communication among neural networks, potentially revealing early markers before they become clinically pronounced. Furthermore, EEG is relatively affordable, non-invasive, and suitable for repeated assessments—important advantages for research aimed at detecting subclinical or progressive alterations in neural dynamics.

## 2 Methods

### 2.1 Participants

The study was conducted on a sample of 100 participants, aged 25–55 years. The inclusion criteria for the study were employee status (currently employed, at least 1.5 years of work experience, active dayshift workers with higher education), right-handedness, and correct or corrected-to-normal vision. Exclusion criteria were pregnancy, addictions, and a history of neurological or psychiatric diseases. Participants were recruited from an initial group of 272 volunteers who responded to an invitation to take part in a scientific project. Its purpose and description were presented via email to organizations, and invitations were posted on university and business social networks. Participants were informed about the financial compensation for the time they dedicated to this EEG study (about 45 EUR). Volunteers were screened using an online study to check if they met the inclusion and exclusion criteria. Selection of participants for the experimental group was based on high burnout symptoms. The controls with low burnout symptoms were matched with an experimental group, taking into account the latter’s demographic characteristics (i.e., gender, age, and education).

### 2.2 Questionnaires

The initial group of 100 participants was selected based on the results of the *Maslach Burnout Inventory—General Survey* (MBI-GS; Maslach et al., 1996). The MBI-GS measures three dimensions of burnout: exhaustion (5 items), cynicism (5 items), and professional efficacy (6 items). It consists of 16 items rated on a 7-point scale ranging from 0 “never” to 6 “every day.” Cronbach’s *α* coefficients based on the sample were α_exhaustion_ = 0.92, α_cynicism_ = 0.91, and α_efficacy_ = 0.89, indicating excellent psychometric characteristics. Participants who scored >3 in exhaustion and cynicism, and < 3 in professional efficacy were identified as a burnout subject. Control group consisted of participants who scored <3 in exhaustion and cynicism, and > 3 in professional efficacy.

To control depressive symptoms the *Beck Depression Inventory* (BDI; Beck et al., 1988) was used. The BDI measures the severity of depressive symptoms. It comprises of 21 items with four response options for each item, rated from 0 to 3 points. Each item reflects specific depression symptom (e.g., sadness, sleep problems). Responses reference the severity of symptoms. Scores range from 0 to 63, with higher scores indicating greater severity of depression symptoms. The symptoms are divided in 4 categories: minimal (scores <10), mild (10–18), moderate (19–29) and severe (30–63) (Beck et al., 1988). Cronbach’s α for the BDI in this sample was 0.90.

### 2.3 Experimental procedure

The protocol of the study was approved by the Bioethics Commission at Jagiellonian University and was carried out in accordance with the recommendations of the APA Ethics Code. Each participant gave written informed consent and was paid for their participation.

The first stage of the study was based on questionnaire survey that included: MBI-GS and BDI. Resting-state EEG was recorded under two conditions: “eyes-open” (EO) and “eyes-closed” (EC). Subjects were instructed to remain as still as possible during the recording period and to react to prerecorded audio commands. In the EO condition, participants were asked to focus on the fixation point positioned in the center of the screen. In the EC condition, participants were instructed to close their eyes and relax until the next audio command. The instructions to “open eyes” and “close eyes” alternated every minute, three times each. In total, the recording lasted 6 min, comprising 3 min of the EO condition and 3 min of the EC condition.

### 2.4 EEG analysis

Continuous dense-array EEG data were collected from 256 channels (HydroCel Geodesic Sensor Net, EGI System 300; Electrical Geodesic Inc., OR, USA), with a sampling rate of 250 Hz, a 0.01–100 Hz band-pass filter, and a vertex electrode as a reference. Data were recorded using NetStation Software (Version 4.5.1, Electrical Geodesic Inc., OR, USA). The impedance for all electrodes was maintained below 50 kΩ. Offline data preprocessing and analysis were conducted using the open-source EEGLAB toolbox (Delorme and Makeig, 2004) and the open-source Python package CUSIGNAL (NumPy Python package).^1^ Before the preprocessing steps, facial channels were removed; thus, further analysis was performed on 224 channels. Data were digitally filtered to remove frequencies below 0.5 Hz and above 35 Hz. Bad channels were automatically removed based on kurtosis measures, using a threshold of 5 standard deviations, and the data was re-referenced to the average signal. Subsequently, continuous data underwent visual inspection to manually identify and remove channels or time epochs containing high-amplitude, high-frequency muscle noise, and other irregular artifacts.

Independent component analysis (ICA) was employed to eliminate artifacts from the data. Given the extensive number of channels, EEG data decomposition using the Infomax algorithm was preceded by Principal Component Analysis (PCA). Fifty independent components were extracted and visually inspected for each subject. Components exhibiting spatiotemporal patterns indicative of blinks, heart rate, saccades, muscle artifacts, or bad channels were excluded. In addition, missing channels were interpolated, and ICA weights were recalculated.

The data were segmented into EO and EC conditions by concatenating respective epochs. To mitigate the influence of auditory event-related potentials on the analysis results, the initial 4 s of each epoch were disregarded. Each open and closed recordings consisted of 3 epochs of 56 s length. We chose a relatively long epoch length based on evidence that quantitative EEG metrics (including coherence) achieve reliable estimates with epochs of 40 s or longer, with marginal improvements beyond this threshold (Gudmundsson et al., 2007). Longer epochs also improve the stability of functional connectivity networks by averaging out the high variability observed over shorter timescales, allowing stable “core” network structures to emerge (Chu et al., 2012).

### 2.5 Statistical analysis

For each subject, the magnitude squared coherence was calculated for each pair of channels using Welch’s method (Welch, 1967):


$$C_{xy}=\frac{{\left|P_{xy}\right|}^{2}}{P_{xx}\cdot P_{yy}}$$

where $P_{xx}$ and $P_{yy}$ are power spectral density estimates of channels X and Y, and $P_{xy}$ is the cross-spectral density estimate of channels X and Y (Welch, 1967). Coherence is used to determine functional connectivity in brain networks. A higher coherence value corresponds to more synchronization between EEG channels and hence stronger functional connections between them. We chose a threshold of 0.5 for coherence to emphasize meaningful connectivity patterns between regions. Further, referring to Adamovich et al.’s (2022) findings on different thresholds in resting-state EEG studies, we extracted stronger connectivity using thresholds of 0.7 to highlight robust connections while filtering out weaker ones.

Mean coherence values for the delta (1–4 Hz), theta (4–7.5 Hz), theta1 (4–6 Hz), theta2 (6–7.5 Hz), alpha (7.5–13 Hz) (Ergenoglu et al., 2004), alpha1 (7.5–9.5 Hz), alpha2 (9.5–11 Hz), alpha3 (11–13 Hz) (Anokhin et al., 2006; Everhart and Demaree, 2003; Iznak et al., 2021), beta (13–35 Hz) (Jensen et al., 2005; Neuper and Pfurtscheller, 2001), beta1 (13–20 Hz) and beta2 (20–35 Hz) (Babiloni et al., 2014) frequency bands were calculated for each subject.

The significance of differences in coherence values between burnout and control groups was assessed for each pair of channels within each frequency band, separately for the eyes-open and eyes-closed conditions. Statistical comparisons were performed with the Mann–Whitney U test; these were subsequently corrected for multiple comparisons using the Benjamini-Hochberg false discovery rate (FDR) method.

## 3 Results

The study sample consisted of 98 participants, selected from the initial group on the basis of the quality of the EEG recordings. The non-clinical burnout group (n = 49; 30 women) had high scores on the exhaustion and cynicism subscales, and low scores on the self-efficacy subscale (see Table 1). Inversely, the control group (*n* = 49; 29 women) had lower scores on the exhaustion and cynicism subscales, and higher scores on the self-efficacy subscale. The depression symptoms were significantly higher in the burnout group but were identified as mild; in the control group, minimal depression symptoms were observed.

**Table 1:** The means (M) and standard deviations (SD) for the burnout and control groups on age, burnout (exhaustion, cynicism, efficacy), and depressive symptoms, work-life areas, and independent-sample *t*-test between burnout and controls.

	Burnout (*n* = 49)	Control (*n* = 49)	*t-*value (df = 96)
	M (SD)	M (SD)	
Age	34.94 (8.45)	37.37 (7.63)	−1.49 *n.s.*
MBI-GS			
Exhaustion	4.13 (1.00)	1.93 (0.75)	−12.33***
Cynicism	4.02 (0.87)	1.44 (0.65)	−16.55***
Efficacy	3.37 (1.13)	4.60 (0.63)	6.62***
BDI			
Depression	14.04 (7.61)	4.76 (4.72)	−7.26***

^***^p < 0.001; n.s., not significant.

**Figure 1:**
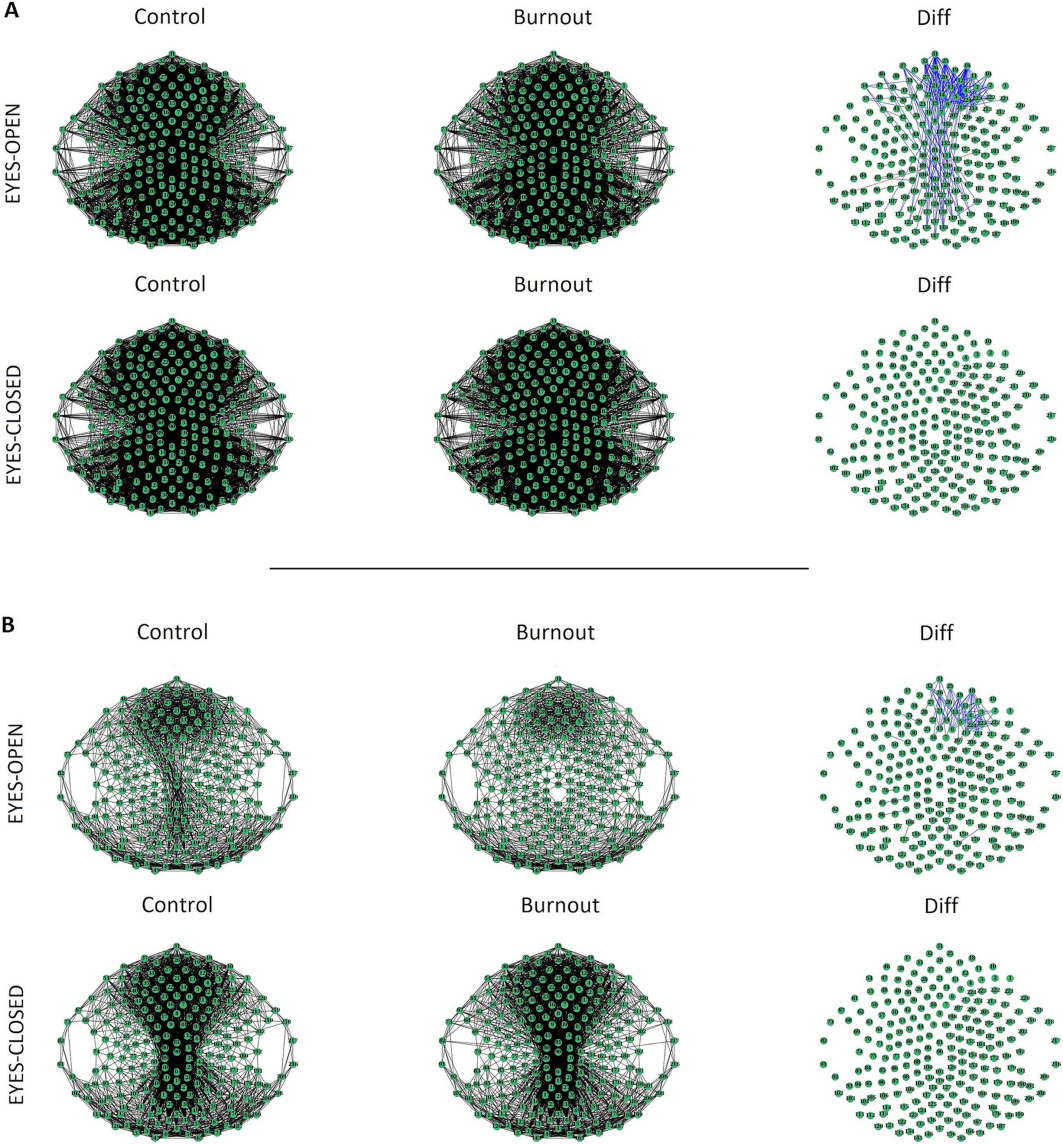
Coherence analysis for the alpha3 band in the control (left) and burnout (middle) groups for the eyes-open (top) and eyes-closed (bottom) conditions. Channels are connected by a black line if the median coherence is above the 0.5 **(A)** or the 0.7 **(B)** threshold. Difference (Diff) between groups (right) is marked by a blue line if median coherence in the burnout group is weaker than in the control group.

The results of the coherence analysis revealed significant differences between the burnout and control groups only in the alpha3 sub-band (11–13 Hz) in the eyes-open condition (Figure 1). No significant differences were observed for the other frequencies.

Compared with the controls, a significant decrease in connectivity in alpha-3 was observed in the frontal and midline areas in the burnout group in the open-eyes-condition (at threshold 0.5), with a particularly notable decrease in the right frontal area. At the more robust 0.7 threshold, this significant connectivity decrease in the right frontal area was still observed. Complete statistics for significant results are presented in Supplementary Table S1A for threshold 0.5 and Supplementary Table S1B for threshold 0.7. No differences between groups were observed in alpha3 sub-bands in the eyes-closed condition. Furthermore, the analysis revealed no differences in the delta, theta, alpha1, alpha2, or beta sub-bands between the burnout group and the control group.

## 4 Discussion

The present study is based on resting-state analysis, which – although it assesses the state of relaxing activity – has been found to be sufficient to reveal significant differences between healthy controls and a variety of disorders such as Alzheimer’s disease and mild cognitive impairment (Meghdadi et al., 2021), depression (de Aguiar Neto and Rosa, 2019), autism spectrum disorder (Heunis et al., 2016), attention deficit-hyperactivity disorder (ADHD), obsessive compulsive disorder (OCD), and schizophrenia (e.g., Newson and Thiagarajan, 2019). Resting-state data are considered a robust indicator of brain functioning, including cognitive performance (Deco et al., 2011). Some studies even suggest that assessing or predicting states such as mental fatigue (Li et al., 2020) and vulnerability to depression (Kaushik et al., 2023) is more effective when conducted using resting-state rather than task-based data.

Previous EEG studies on burnout syndrome have shown interesting spectral analysis findings in the resting state, but only in the eyes-open condition (Luijtelaar et al., 2010; Golonka et al., 2019a). On the other hand, there are several preliminary studies on resting-state functional connectivity in emotional exhaustion/burnout among students, but unfortunately limited only to the eyes-closed condition (i.e., Harmatiuk et al., 2023; Tement et al., 2016; Tukaiev et al., 2012, 2022). The presented research is the first study to analyse resting-state brain functional connectivity based on EEG coherence in occupational burnout in a sample of working adults, analyzing both eyes-open and eyes-closed conditions. The analysis revealed prominent differences between burnout and controls in the alpha3 sub-band, thus indicating a significant decrease in brain activity in the burnout group in frontal and midline areas. It is worth emphasizing that decreased connectivity in burnout was observed only in the eyes-open condition and was strongest in the right frontal areas. These findings shed light on the dynamic functional connectivity changes associated with burnout syndrome and provide insights into the neurobiological underpinnings of this phenomenon.

The results of this study indicate that eyes-open conditions should be considered in future studies on burnout using a resting-state paradigm. This is in line with Luijtelaar et al. (2010), who observed a significant difference in alpha frequency between burnout and controls in the eyes-open condition, while no differences were observed in the eyes-closed condition. Similarly, a spectral analysis of resting-state EEG showed that burnout may be distinguished from controls by lower alpha power in burnout only in the eyes-open condition (Golonka et al., 2019a). As was shown by Han et al. (2023), the eyes-closed condition is linked with general brain integration and increased connectivity between most brain networks; in contrast, increased connectivity is observed within the default mode network and between the salience and visual networks in the eyes-open condition. According to Zhang et al. (2019) and Han et al. (2023), the eyes-closed condition is associated with intrinsic activity and self-related mental processes, while the eyes-open condition is associated with externally oriented attention. In this context, it may be assumed that externally oriented attention may be impaired in burnout subjects.

The reduced functional connectivity in the alpha frequency that was observed in this study has also been noticed in mild cognitive impairments (MCI), dementia, ADHD and fatigue (e.g., Basharpoor et al., 2021; Fischer et al., 2023; Gurja et al., 2022; Kim et al., 2024; Lejko et al., 2020; Murias et al., 2007; Xu et al., 2014). However, in their systematic review and meta-analysis, Lejko et al. (2020) indicate that individuals with MCI may reveal either decreased or increased alpha functional connectivity as compared to healthy controls. Thus, it is important not to treat a single indicator as a discriminant factor for complex syndromes or disorders due to diverse methodological problems. Lejko et al. (2020) suggest that inconsistent results in alpha connectivity may be caused by spurious connections due to a small number of electrodes without spatial filters. In this context, analysis based on dense-array EEG may particularly contribute to research on functional connectivity in burnout syndrome.

The presented analyses indicate weaker functional connectivity in the right frontal area. This may suggest significant frontal asymmetry in brain coherence in the burnout group. The first EEG study that analyzed asymmetry in frontal alpha connectivity was presented by Imperatori et al. (2019), who introduced a new neurophysiological Frontal Alpha Connectivity Asymmetry Index (FACA-I). In their analysis on a non-clinical sample of university students, they found that lower connectivity between the right medial Prefrontal Cortex (mPFC) and the subgenual Anterior Cingulate Cortex (sgACC) is significantly associated with cognitive/affective and somatic symptoms of depression. Interestingly, Imperatori et al. (2019) showed that FACA-I may be a better index of depressive symptoms than Frontal Alpha Asymmetry (FAA). The weakness of FAA as a diagnostic marker in depression was also emphasized by Van Der Vinne et al. (2017), who indicated the necessity to address the “gender × age × depression severity” interaction. Imperatori et al. (2019) found that when controlling for anxiety, gender and age, significant associations between depression symptoms and FACA-I were still observed, but no significant correlation was found with FAA, thus supporting the findings presented by Van Der Vinne et al. (2017). Moreover, FACA-I did not correlate with FAA, which suggests that these indexes describe different brain mechanisms. Thus, observations regarding lower frontal alpha connectivity in burnout subjects versus controls may be particularly promising and indicate important directions for further neurophysiological research on burnout syndrome.

Besides a decline in alpha frontal functional connectivity, a significant decrease in alpha3 connectivity in the eyes-open condition was observed in midline areas. Reduced connectivity in some cortical midline structures (i.e., isthmus cingulate and precuneus) have previously been linked with rumination in PTSD and depression (Philippi et al., 2020). This perspective may be particularly valuable due to maladaptive cognitive emotion regulation strategies that are typical of burnout syndrome, such as rumination, catastrophizing and self-blame (Głaziewicz and Golonka, 2024). Current findings on decreased midline functional connectivity are based mainly on task-oriented fMRI studies, but some of them may be particularly inspiring for the interpretation of our results. For example, Doering et al. (2012) showed a relationship between midline cortical structures, personality functioning and identity integration. However, these findings represent a completely new perspective for burnout syndrome and should be treated with caution. Further EEG research using the resting-state paradigm is needed to verify our findings and to define some psychological variables that could direct the interpretation of achieved outcomes. Nevertheless, the fact that our study revealed the same reduced connectivity in midline brain areas as was observed in studies on rumination and cognitive impairments indicates that an interesting direction for further EEG studies would be to focus on cognitive functions, emotion regulation, and coping strategies in burnout syndrome.

The results may represent an important insight into differentiating burnout from depression as our study on burnout individuals did not reveal all the same findings as have been reported by many previous studies on depression. For example, while this study indicated decreased functional connectivity in the alpha3 sub-band, some findings on depression indicate increased connectivity in the delta, theta and alpha frequency bands (e.g., Fingelkurts et al., 2007; Leuchter et al., 2012). As pointed out by Dell’Acqua et al. (2021), this increased functional connectivity within the theta and alpha bands, as is also observed in dysphoria, may be considered a marker of the “idling brain.” However, such patterns were not observed in burnout in our analysis. Although FAA has been reported in some studies on depression (e.g., Allen and Reznik, 2015) and on stress and difficulties in emotion regulation (e.g., Goodman et al., 2013; Zhang et al., 2020), it has not been found in previous burnout research (Luijtelaar et al., 2010; Golonka et al., 2019a). In light of the meta-analysis presented by Miljevic et al. (2023), while many EEG functional connectivity studies on depression have observed differences in the alpha, theta, and beta frequencies, drawing definitive conclusions regarding the direction of these variances has been challenging due to significant disparities in study designs and methodologies. Miljevic et al.’s most consistent finding indicated higher connectivity in the frontal regions in the alpha frequency band and lower connectivity in the parieto-occipital regions in the alpha band. However, it is important to emphasize that most of the EEG studies in the resting-state paradigm analyzed by Miljevic et al. (2023) were conducted in the eyes-closed condition, but no significant differences were detected in our study in this condition. In comparisons between depressed subjects and healthy controls in the resting-state paradigm, only a few EEG studies have also used an eyes-open condition. For example, Bailey et al. (2019) did not observe any differences in the alpha and theta bands; Duan et al. (2020) showed increased connectivity in the alpha band in occipital regions and in the beta band in parietal and central regions; Markovska-Simoska et al. (2018) observed increased alpha and beta connectivity in central areas and decreased connectivity in the delta band in occipito-parietal regions; Whitton et al. (2018) found significant differences only in the beta bands. Thus, regarding the decreased connectivity in the high alpha band in the frontal and midline areas in the eyes-open condition that was observed in our study, no similarities between EEG functional connectivity in burnout and depression may be confirmed.

These findings contribute to the growing body of knowledge on the neural correlates of burnout, fostering a deeper understanding of its underlying mechanisms. Results confirm previous neuroimaging studies that showed functional changes in frontal areas in burnout subjects (Abe et al., 2022; Blix et al., 2013; Golkar et al., 2014; Grossi et al., 2015; Savic et al., 2018; Sandström et al., 2011; Tei et al., 2014). Referring to previous EEG studies on burnout, the results confirm significant changes in alpha band (Luijtelaar et al., 2010; Golonka et al., 2019a; Harmatiuk et al., 2023; Tement et al., 2016). The significance of this research extends beyond the academic realm, offering valuable insights for clinicians, psychologists, and organizations concerned with mental health and wellbeing in high-stress environments. The evidence of neurobiological changes associated with burnout reinforces the need for prevention policies and early interventions in the workplace. This could include the implementation of mental wellbeing programs, adjustments in workloads, and the promotion of healthier work environments. Results suggest that workplace interventions aimed at reducing burnout could benefit from focusing on relaxation techniques and mindfulness (Cohen et al., 2023), which have been associated with changes in brain functional connectivity (Yue et al., 2023; Rathore et al., 2022). Programs that promote stress reduction and enhance mental wellbeing could potentially reverse or mitigate the connectivity changes observed in burnout. Identifying specific patterns of functional connectivity in burnout syndrome provides a potential direction for developing targeted interventions or preventive strategies, such as targeted neuromodulation (e.g., repetitive transcranial magnetic stimulation, TMS), which can be directed to specific brain areas that show connectivity dysfunctions. Ultimately, this research may have implications for the development of personalized approaches to alleviate and prevent burnout syndrome, thereby enhancing employees’ mental health and their efficacy in various professional settings.

### 4.1 Limitations of the study and implications for future research

In this study, the burnout group was non-clinical due to the inclusion criteria and was characterized by mild depression symptoms. As many findings suggest, burnout is associated with higher levels of depression (e.g., Schonfeld et al., 2019; Van Dam, 2016), therefore the overlapping effect with depression symptoms should be taken into account. In future studies, it may be valuable to compare two groups of burnout employees with mild versus moderate/severe depression symptoms. Incorporating comprehensive clinical evaluations encompassing assessments for anxiety, depression, and other psychiatric conditions can facilitate the delineation of burnout-specific neural markers from those attributed to comorbid mental health disorders. Future studies could more thoroughly explore the differences and similarities in functional connectivity between burnout, depression, anxiety, chronic fatigue (CF), and other stress-related syndromes in order to better discriminate these conditions based on neural biomarkers.

It is also important to recognize the limitations of study protocol and coherence analysis. The protocol of three 1-min recordings for eyes-open and eyes-closed conditions might interfere with the resting-state condition and limit the stability of brain activity measurements. While this approach encourages participants to actively engage with the transition between eyes-open and eyes-closed states, future studies should consider longer recording times to ensure stable brain activity. Although coherence provides valuable insights into functional connectivity, it measures linear synchronization between two signals and may not fully capture the complex non-linear interactions between brain regions. Complementary methods and simultaneous tests using dEEG and, e.g., fMRI could provide a more comprehensive understanding of neural interactions. Future research endeavors could also adopt a longitudinal study design to elucidate whether the observed changes in functional connectivity are reversible through interventions or changes in the occupational environment. Given that differences were detected only in the eyes-open condition, it is recommended to include the eyes-open condition in subsequent analyses of functional connectivity in the resting state in burnout.

The discussion could further elaborate on the interpretation of functional connectivity differences found in the alpha3 sub-band and on how these relate to specific cognitive functions that are affected in burnout. For instance, alpha source connectivity has been associated with mild cognitive impairments in the eyes-closed resting-state condition (Babiloni et al., 2018), suggesting that the patterns observed in this study might be linked to cognitive deficits that are frequently reported by individuals with burnout. Integrating task-based EEG assessments alongside resting-state investigations could enrich our comprehension of brain function in burnout, offering a holistic perspective on neural dynamics under varying cognitive demands. Exploring the relationship between measures of functional connectivity and cognitive performance could shed light on how observed neural changes manifest in everyday cognitive functioning among individuals experiencing burnout (Newson and Thiagarajan, 2019). In this context, analysis of functional connectivity may be a particularly promising direction for future studies on burnout syndrome.

## 5 Conclusion

This study provides a comprehensive examination of resting-state functional brain connectivity in individuals experiencing occupational burnout compared with controls. The patterns observed in the eyes-open resting-state condition suggest a potential neurobiological basis for burnout syndrome that is characterized by decreased functional connectivity in the alpha3 sub-band (11–13 Hz) in the frontal and midline brain areas, with the strongest effect in the right frontal area. The results indicate that the eyes-open condition is recommended in further resting-state protocols on burnout syndrome.

## Data Availability

The raw data supporting the conclusions of this article will be made available by the authors, without undue reservation.

## Footnotes

^1^ https://numpy.org/

## References

[ref1] Abe K. Tei S. Takahashi H. Fujino J. (2022). Structural brain correlates of burnout severity in medical professionals: a voxel-based morphometric study. Neurosci. Lett. 772:136484. doi: 10.1016/j.neulet.2022.136484, PMID: 35108589 PMC9758014

[ref2] Adamovich T. Zakharov I. Tabueva A. Malykh S. (2022). The thresholding problem and variability in the EEG graph network parameters. Sci. Rep. 12:18659. doi: 10.1038/s41598-022-22079-2, PMID: 36333413 PMC9636266

[ref3] Allen J. J. Reznik S. J. (2015). Frontal EEG asymmetry as a promising marker of depression vulnerability: summary and methodological considerations. Curr. Opin. Psychol. 4, 93–97. doi: 10.1016/j.copsyc.2014.12.017, PMID: 26462291 PMC4599354

[ref4] Anokhin A. P. Heath A. C. Myers E. (2006). Genetic and environmental influences on frontal EEG asymmetry: a twin study. Biol. Psychol. 71, 289–295. doi: 10.1016/j.biopsycho.2005.06.004, PMID: 16054745 PMC2174210

[ref5] Arias-Carrión O. Stamelou M. Murillo-Rodríguez E. Menéndez-González M. Pöppel E. (2010). Dopaminergic reward system: a short integrative review. Int. Arch. Med. 3:24. doi: 10.1186/1755-7682-3-24, PMID: 20925949 PMC2958859

[ref6] Babiloni C. Del Percio C. Lizio R. Marzano N. Infarinato F. Soricelli A.. (2014). Cortical sources of resting state electroencephalographic alpha rhythms deteriorate across time in subjects with amnesic mild cognitive impairment. Neurobiol. Aging 35, 130–142. doi: 10.1016/j.neurobiolaging.2013.06.019, PMID: 23906617

[ref7] Babiloni C. Del Percio C. Lizio R. Noce G. Lopez S. Soricelli A.. (2018). Functional cortical source connectivity of resting state electroencephalographic alpha rhythms shows similar abnormalities in patients with mild cognitive impairment due to Alzheimer’s and Parkinson’s diseases. Clin. Neurophysiol. 129, 766–782. doi: 10.1016/j.clinph.2018.01.009, PMID: 29448151

[ref8] Bailey N. W. Hoy K. E. Rogasch N. C. Thomson R. H. McQueen S. Elliot D.. (2019). Differentiating responders and non-responders to rTMS treatment for depression after one week using resting EEG connectivity measures. J. Affect. Disord. 242, 68–79. doi: 10.1016/j.jad.2018.08.058, PMID: 30172227

[ref9] Basharpoor S. Heidari F. Molavi P. (2021). EEG coherence in theta, alpha, and beta bands in frontal regions and executive functions. Appl. Neuropsychol. Adult 28, 310–317. doi: 10.1080/23279095.2019.1632860, PMID: 31282216

[ref10] Beck A. T. Steer R. A. Carbin M. G. (1988). Psychometric properties of the beck depression inventory: twenty-five years of evaluation. Clin. Psychol. Rev. 8, 77–100. doi: 10.1016/0272-7358(88)90050-5

[ref11] Blackburn B. Chan T. Cherot E. Freeman R. B. Hu X. Matt E.. (2023). Beyond burnout: From measuring to forecasting. Cambridge, MA: National Bureau of Economic Research.

[ref12] Blix E. Perski A. Berglund H. Savic I. (2013). Long-term occupational stress is associated with regional reductions in brain tissue volumes. PLoS One 8:e64065. doi: 10.1371/journal.pone.0064065, PMID: 23776438 PMC3679112

[ref13] Bowyer S. M. (2016). Coherence a measure of the brain networks: past and present. Neuropsychiatr. Electrophysiol. 2, 1–12. doi: 10.1186/s40810-015-0015-7

[ref14] Canu I. G. Mesot O. Györkös C. Mediouni Z. Mehlum I. S. Bugge M. D. (2019). Burnout syndrome in Europe: towards a harmonized approach in occupational health practice and research. Ind. Health 57, 745–752. doi: 10.2486/indhealth.2018-0159, PMID: 30814391 PMC6885602

[ref15] Chu C. J. Kramer M. A. Pathmanathan J. Bianchi M. T. Westover M. B. Wizon L.. (2012). Emergence of stable functional networks in long-term human electroencephalography. J. Neurosci. 32, 2703–2713. doi: 10.1523/JNEUROSCI.5669-11.2012, PMID: 22357854 PMC3361717

[ref16] Cohen C. Pignata S. Bezak E. Tie M. Childs J. (2023). Workplace interventions to improve well-being and reduce burnout for nurses, physicians and allied healthcare professionals: a systematic review. BMJ Open 13:e071203. doi: 10.1136/bmjopen-2022-071203, PMID: 37385740 PMC10314589

[ref17] de Aguiar Neto F. S. Rosa J. L. G. (2019). Depression biomarkers using non-invasive EEG: a review. Neurosci. Biobehav. Rev. 105, 83–93. doi: 10.1016/j.neubiorev.2019.07.021, PMID: 31400570

[ref18] de Andrade A. P. M. Amaro E. Jr. Farhat S. C. L. Schvartsman C. (2016). Higher burnout scores in paediatric residents are associated with increased brain activity during attentional functional magnetic resonance imaging task. Acta Paediatr. 105, 705–713. doi: 10.1111/apa.13371, PMID: 26896193

[ref19] Deco G. Jirsa V. K. McIntosh A. R. (2011). Emerging concepts for the dynamical organization of resting-state activity in the brain. Nat. Rev. Neurosci. 12, 43–56. doi: 10.1038/nrn2961, PMID: 21170073

[ref20] Dell’Acqua C. Ghiasi S. Benvenuti S. M. Greco A. Gentili C. Valenza G. (2021). Increased functional connectivity within alpha and theta frequency bands in dysphoria: a resting-state EEG study. J. Affect. Disord. 281, 199–207. doi: 10.1016/j.jad.2020.12.015, PMID: 33326893

[ref21] Delorme A. Makeig S. (2004). EEGLAB: an open source toolbox for analysis of single-trial EEG dynamics including independent component analysis. J. Neurosci. Methods 134, 9–21. doi: 10.1016/j.jneumeth.2003.10.009, PMID: 15102499

[ref22] Doering S. Enzi B. Faber C. Hinrichs J. Bahmer J. Northoff G. (2012). Personality functioning and the cortical midline structures–an exploratory fMRI study. PLoS One 7:e49956. doi: 10.1371/journal.pone.0049956, PMID: 23189175 PMC3506600

[ref23] Duan L. Duan H. Qiao Y. Sha S. Qi S. Zhang X.. (2020). Machine learning approaches for MDD detection and emotion decoding using EEG signals. Front. Hum. Neurosci. 14:284. doi: 10.3389/fnhum.2020.00284, PMID: 33173472 PMC7538713

[ref24] Durning S. J. Costanzo M. Artino A. R. Jr. Dyrbye L. N. Beckman T. J. Schuwirth L.. (2013). Functional neuroimaging correlates of burnout among internal medicine residents and faculty members. Front. Psych. 4:131. doi: 10.3389/fpsyt.2013.00131, PMID: 24133462 PMC3796712

[ref25] Ergenoglu T. Demiralp T. Bayraktaroglu Z. Ergen M. Beydagi H. Uresin Y. (2004). Alpha rhythm of the EEG modulates visual detection performance in humans. Cogn. Brain Res. 20, 376–383. doi: 10.1016/j.cogbrainres.2004.03.009, PMID: 15268915

[ref26] Everhart D. E. Demaree H. A. (2003). Low alpha power (7.5–9.5 Hz) changes during positive and negative affective learning. Cognitive, affective, & behavioral. Neuroscience 3, 39–45. doi: 10.3758/CABN.3.1.39, PMID: 12822597

[ref27] Fingelkurts A. Fingelkurts A. Rytsälä H. Suominen K. Isometsä E. Kähkönen S. (2007). Impaired functional connectivity at EEG alpha and theta frequency bands in major depression. Hum. Brain Mapp. 28, 247–261. doi: 10.1002/hbm.20275, PMID: 16779797 PMC6871285

[ref28] Fischer M. H. F. Zibrandtsen I. C. Høgh P. Musaeus C. S. (2023). Systematic review of EEG coherence in Alzheimer’s disease. J. Alzheimers Dis. 91, 1261–1272. doi: 10.3233/JAD-220508, PMID: 36641665

[ref29] Friberg T. (2009). Burnout: from popular culture to psychiatric diagnosis in Sweden. Cult. Med. Psychiatry 33, 538–558. doi: 10.1007/s11013-009-9149-z, PMID: 19795101

[ref30] Friston K. J. (2011). Functional and effective connectivity: a review. Brain Connect. 1, 13–36. doi: 10.1089/brain.2011.0008, PMID: 22432952

[ref31] Gavelin H. M. Neely A. S. Dunås T. Eskilsson T. Järvholm L. S. Boraxbekk C. J. (2020). Mental fatigue in stress-related exhaustion disorder: structural brain correlates, clinical characteristics and relations with cognitive functioning. Neuro Image Clin. 27:102337. doi: 10.1016/j.nicl.2020.102337, PMID: 32652491 PMC7348057

[ref32] Głaziewicz K. Golonka K. (2024). When the creative well dries up – burnout syndrome and art block in artists’ sample. Think. Skills Creat. 54:101692. doi: 10.1016/j.tsc.2024.101692

[ref33] Golkar A. Johansson E. Kasahara M. Osika W. Perski A. Savic I. (2014). The influence of work-related chronic stress on the regulation of emotion and on functional connectivity in the brain. PLoS One 9:e104550. doi: 10.1371/journal.pone.0104550, PMID: 25184294 PMC4153588

[ref34] Golonka K. Gawlowska M. Mojsa-Kaja J. Marek T. (2019a). Psychophysiological characteristics of burnout syndrome: resting-state EEG analysis. Bio Med Res. Int. 2019, 1–8. doi: 10.1155/2019/3764354, PMID: 31467886 PMC6701350

[ref35] Golonka K. Mojsa-Kaja J. Blukacz M. Gawłowska M. Marek T. (2019b). Occupational burnout and its overlapping effect with depression and anxiety. Int. J. Occup. Med. Environ. Health 32, 229–244. doi: 10.13075/ijomeh.1896.01323, PMID: 30855601

[ref36] Golonka K. Mojsa-Kaja J. Gawlowska M. Popiel K. (2017a). Cognitive impairments in occupational burnout–error processing and its indices of reactive and proactive control. Front. Psychol. 8:676. doi: 10.3389/fpsyg.2017.00676, PMID: 28507528 PMC5410591

[ref37] Golonka K. Mojsa-Kaja J. Popiel K. Marek T. Gawlowska M. (2017b). Neurophysiological markers of emotion processing in burnout syndrome. Front. Psychol. 8:2155. doi: 10.3389/fpsyg.2017.02155, PMID: 29326619 PMC5736989

[ref38] Goodman R. N. Rietschel J. C. Lo L. C. Costanzo M. E. Hatfield B. D. (2013). Stress, emotion regulation and cognitive performance: the predictive contributions of trait and state relative frontal EEG alpha asymmetry. Int. J. Psychophysiol. 87, 115–123. doi: 10.1016/j.ijpsycho.2012.09.008, PMID: 23022494

[ref39] Grossi G. Perski A. Osika W. Savic I. (2015). Stress-related exhaustion disorder–clinical manifestation of burnout? A review of assessment methods, sleep impairments, cognitive disturbances, and neuro-biological and physiological changes in clinical burnout. Scand. J. Psychol. 56, 626–636. doi: 10.1111/sjop.12251, PMID: 26496458

[ref40] Gudmundsson S. Runarsson T. P. Sigurdsson S. Eiriksdottir G. Johnsen K. (2007). Reliability of quantitative EEG features. Clin. Neurophysiol. 118, 2162–2171. doi: 10.1016/j.clinph.2007.06.018, PMID: 17765604

[ref41] Gurja J. P. Muthukrishnan S. P. Tripathi M. Sharma R. (2022). Reduced resting-state cortical alpha connectivity reflects distinct functional brain dysconnectivity in Alzheimer's disease and mild cognitive impairment. Brain Connect. 12, 134–145. doi: 10.1089/brain.2020.0926, PMID: 34030487

[ref42] Han J. Zhou L. Wu H. Huang Y. Qiu M. Huang L.. (2023). Eyes-open and eyes-closed resting state network connectivity differences. Brain Sci. 13:122. doi: 10.3390/brainsci13010122, PMID: 36672103 PMC9857293

[ref43] Harmatiuk D. Tukaiev S. Popov A. Makarchuk M. (2023). “Burnout-specific changes in brain activity (under way to exhaustion,” in 2023 *Signal Processing Symposium (SPSympo), Karpacz, Poland.* Piscataway, NJ: The Institute of Electrical and Electronics Engineers (IEEE), 57–62.

[ref44] Heinemann L. V. Heinemann T. (2017). Burnout research: emergence and scientific investigation of a contested diagnosis. SAGE Open 7:2158244017697154. doi: 10.1177/2158244017697154

[ref45] Heunis T. M. Aldrich C. de Vries P. J. (2016). Recent advances in resting-state electroencephalography biomarkers for autism spectrum disorder - a review of methodological and clinical challenges. Pediatr. Neurol. 61, 28–37. doi: 10.1016/j.pediatrneurol.2016.03.010, PMID: 27255413

[ref46] Huang M. H. Fan S. Y. Lin I. M. (2023). EEG coherences of the fronto-limbic circuit between patients with major depressive disorder and healthy controls. J. Affect. Disord. 331, 112–120. doi: 10.1016/j.jad.2023.03.055, PMID: 36958482

[ref47] Imperatori C. Farina B. Valenti E. M. Di Poce A. D'Ari S. De Rossi E.. (2019). Is resting state frontal alpha connectivity asymmetry a useful index to assess depressive symptoms? A preliminary investigation in a sample of university students. J. Affect. Disord. 257, 152–159. doi: 10.1016/j.jad.2019.07.034, PMID: 31301617

[ref48] Iznak A. F. Iznak E. V. Damyanovich E. V. Oleichik I. V. (2021). Differences of EEG frequency and spatial parameters in depressive female adolescents with suicidal attempts and non-suicidal self-injuries. Clin. EEG Neurosci. 52, 406–413. doi: 10.1177/1550059421991685, PMID: 33555208

[ref49] Jensen O. Goel P. Kopell N. Pohja M. Hari R. Ermentrout B. (2005). On the human sensorimotor-cortex beta rhythm: sources and modeling. NeuroImage 26, 347–355. doi: 10.1016/j.neuroimage.2005.02.008, PMID: 15907295

[ref50] Kaushik P. Yang H. Roy P. P. van Vugt M. (2023). Comparing resting state and task-based EEG using machine learning to predict vulnerability to depression in a non-clinical population. Sci. Rep. 13:7467. doi: 10.1038/s41598-023-34298-2, PMID: 37156879 PMC10167316

[ref51] Kim M. S. Park S. Park U. Kang S. W. Kang S. Y. (2024). Fatigue in Parkinson’s disease is due to decreased efficiency of the frontal network: quantitative EEG analysis. J. Movem. Disorders 17, 304–312. doi: 10.14802/jmd.24038, PMID: 38853446 PMC11300402

[ref52] Lastovkova A. Carder M. Rasmussen H. M. Sjoberg L. de Groene G. J. Sauni R.. (2018). Burnout syndrome as an occupational disease in the European Union: an exploratory study. Ind. Health 56, 160–165. doi: 10.2486/indhealth.2017-0132, PMID: 29109358 PMC5889935

[ref53] Lejko N. Larabi D. I. Herrmann C. S. Aleman A. Ćurčić-Blake B. (2020). Alpha power and functional connectivity in cognitive decline: a systematic review and meta-analysis. J. Alzheimers Dis. 78, 1047–1088. doi: 10.3233/JAD-200962, PMID: 33185607 PMC7739973

[ref54] Leuchter A. F. Cook I. A. Hunter A. M. Cai C. Horvath S. (2012). Resting-state quantitative electroencephalography reveals increased neurophysiologic connectivity in depression. PLoS One 7:e32508. doi: 10.1371/journal.pone.0032508, PMID: 22384265 PMC3286480

[ref55] Li G. Huang S. Xu W. Jiao W. Jiang Y. Gao Z.. (2020). The impact of mental fatigue on brain activity: a comparative study both in resting state and task state using EEG. BMC Neurosci. 21, 1–9. doi: 10.1186/s12868-020-00569-1, PMID: 32398004 PMC7216620

[ref56] Luijtelaar G. Verbraak M. van den Bunt M. Keijsers G. Arns M. (2010). EEG findings in burnout patients. J. Neuropsychiatry Clin. Neurosci. 22, 208–217. doi: 10.1176/jnp.2010.22.2.208, PMID: 20463115

[ref57] Mano T. Kinugawa K. Ozaki M. Kataoka H. Sugie K. (2022). Neural synchronization analysis of electroencephalography coherence in patients with Parkinson’s disease-related mild cognitive impairment. Clin. Parkinsonism Related Disorders 6:100140. doi: 10.1016/j.prdoa.2022.100140, PMID: 35308256 PMC8928128

[ref58] Markovska-Simoska S. Pop-Jordanova N. Pop-Jordanov J. (2018). Inter-and intra-hemispheric EEG coherence study in adults with neuropsychiatric disorders. Contrib. Macedonian Acad. Sci. Arts 39, 5–19. doi: 10.2478/prilozi-2018-0037, PMID: 30864354

[ref59] Maslach C. Jackson S. E. (1981). The measurement of experienced burnout. J. Organ. Behav. 2, 99–113. doi: 10.1002/job.4030020205

[ref60] Maslach C. Jackson S. E. Leiter M. P. (1996). MBI: Maslach burnout inventory. Sunnyvale, CA: CPP, Incorporated.

[ref61] Maslach C. Schaufeli W. B. Leiter M. P. (2001). Job burnout. Annu. Rev. Psychol. 52, 397–422. doi: 10.1146/annurev.psych.52.1.397, PMID: 11148311

[ref62] Meghdadi A. H. Stevanović Karić M. McConnell M. Rupp G. Richard C. Hamilton J.. (2021). Resting state EEG biomarkers of cognitive decline associated with Alzheimer’s disease and mild cognitive impairment. PLoS One 16:e0244180. doi: 10.1371/journal.pone.0244180, PMID: 33544703 PMC7864432

[ref63] Miljevic A. Bailey N. W. Murphy O. W. Perera M. P. N. Fitzgerald P. B. (2023). Alterations in EEG functional connectivity in individuals with depression: a systematic review. J. Affect. Disord. 328, 287–302. doi: 10.1016/j.jad.2023.01.126, PMID: 36801418

[ref64] Murias M. Swanson J. M. Srinivasan R. (2007). Functional connectivity of frontal cortex in healthy and ADHD children reflected in EEG coherence. Cereb. Cortex 17, 1788–1799. doi: 10.1093/cercor/bhl089, PMID: 17023555 PMC2084383

[ref65] Neuper C. Pfurtscheller G. (2001). Event-related dynamics of cortical rhythms: frequency-specific features and functional correlates. Int. J. Psychophysiol. 43, 41–58. doi: 10.1016/S0167-8760(01)00178-7, PMID: 11742684

[ref66] Newson J. J. Thiagarajan T. C. (2019). EEG frequency bands in psychiatric disorders: a review of resting state studies. Front. Hum. Neurosci. 12:521. doi: 10.3389/fnhum.2018.00521, PMID: 30687041 PMC6333694

[ref67] Philippi C. L. Pessin S. Reyna L. Floyd T. Bruce S. E. (2020). Cortical midline structures associated with rumination in women with PTSD. J. Psychiatr. Res. 131, 69–76. doi: 10.1016/j.jpsychires.2020.09.001, PMID: 32942190 PMC7669571

[ref68] Rathore M. Verma M. Nirwan M. Trivedi S. Pai V. (2022). Functional connectivity of prefrontal cortex in various meditation techniques - a Mini-review. Int. J. Yoga 15, 187–194. doi: 10.4103/ijoy.ijoy_88_22, PMID: 36949839 PMC10026337

[ref69] Sandström A. Peterson J. Sandström E. Lundberg M. Nystrom I. L. R. Nyberg L.. (2011). Cognitive deficits in relation to personality type and hypothalamic-pituitary-adrenal (HPA) axis dysfunction in women with stress-related exhaustion. Scand. J. Psychol. 52, 71–82. doi: 10.1111/j.1467-9450.2010.00844.x, PMID: 20964695

[ref71] Savic I. Perski A. Osika W. (2018). MRI shows that exhaustion syndrome due to chronic occupational stress is associated with partially reversible cerebral changes. Cereb. Cortex 28, 894–906. doi: 10.1093/cercor/bhw413, PMID: 28108490

[ref72] Schaufeli W. B. (2018). Work engagement in Europe: relations with national economy, governance and culture. Organ. Dyn. 47, 99–106. doi: 10.1016/j.orgdyn.2018.01.003

[ref73] Schonfeld I. S. Verkuilen J. Bianchi R. (2019). Inquiry into the correlation between burnout and depression. J. Occup. Health Psychol. 24, 603–616. doi: 10.1037/ocp0000151, PMID: 30945922

[ref74] Shang Y. Yang Y. Zheng G. Zhao Z. Wang Y. Yang L.. (2022). Aberrant functional network topology and effective connectivity in burnout syndrome. Clin. Neurophysiol. 138, 163–172. doi: 10.1016/j.clinph.2022.03.014, PMID: 35453016

[ref9001] Sokka L. Huotilainen M. Leinikka M. Korpela J. Henelius A. Alain C.. (2014). Alterations in attention capture to auditory emotional stimuli in job burnout: an event-related potential study. Int. J. Psychophysiol. 94, 427–436. doi: 10.1016/j.ijpsycho.2014.11.001 25448269

[ref9002] Sokka L. Leinikka M. Korpela J. Henelius A. Lukander J. Pakarinen S.. (2017). Shifting of attentional set is inadequate in severe burnout: evidence from an event-related potential study. Int. J. Psychophysiol. 112, 70–79. doi: 10.1016/j.ijpsycho.2016.12.004 27988179

[ref75] Srinivasan R. (1999). Spatial structure of the human alpha rhythm: global correlation in adults and local correlation in children. Clin. Neurophysiol. 110, 1351–1362. doi: 10.1016/S1388-2457(99)00080-2, PMID: 10454270

[ref76] Tei S. Becker C. Kawada R. Fujino J. Jankowski K. F. Sugihara G.. (2014). Can we predict burnout severity from empathy-related brain activity? Transl. Psychiatry 4:e393. doi: 10.1038/tp.2014.34, PMID: 24893064 PMC4080316

[ref77] Tement S. Pahor A. Jaušovec N. (2016). EEG alpha frequency correlates of burnout and depression: the role of gender. Biol. Psychol. 114, 1–12. doi: 10.1016/j.biopsycho.2015.11.005, PMID: 26631352

[ref78] Tukaiev S. Harmatiuk D. Popov A. Makarchuk M. (2022). Towards EEG biomarkers of emotional burnout syndrome: gender related variations in functional connectivity under resistance stage formation. Eur. Psychiatry 65, S300–S301. doi: 10.1192/j.eurpsy.2022.766

[ref79] Tukaiev S. Krizhanovskiy S. Zima I. Filimonova N. Radchuk O. Cherninskiy A.. (2012). Gender-related differences in spatial synchronization in EEG in burnout students. Psychophysiology 49:S120.

[ref80] Van Dam A. (2016). Subgroup analysis in burnout: relations between fatigue, anxiety, and depression. Front. Psychol. 7:173953. doi: 10.3389/fpsyg.2016.00090, PMID: 26869983 PMC4740380

[ref81] Van Der Vinne N. Vollebregt M. A. Van Putten M. J. Arns M. (2017). Frontal alpha asymmetry as a diagnostic marker in depression: fact or fiction? A meta-analysis. Neuroimage: Clin. 16, 79–87. doi: 10.1016/j.nicl.2017.07.006, PMID: 28761811 PMC5524421

[ref82] Welch P. (1967). The use of fast Fourier transform for the estimation of power spectra: a method based on time averaging over short, modified periodograms. IEEE Trans. Audio Electroacoust. 15, 70–73. doi: 10.1109/TAU.1967.1161901

[ref83] Whitton A. E. Deccy S. Ironside M. L. Kumar P. Beltzer M. Pizzagalli D. A. (2018). Electroencephalography source functional connectivity reveals abnormal high-frequency communication among large-scale functional networks in depression. Biolo. Psychiatr. Cognit. Neurosci. Neuroimaging 3, 50–58. doi: 10.1016/j.bpsc.2017.07.001, PMID: 29397079 PMC5801763

[ref84] World Health Organization (2019). 11th revision of the international classification of diseases (ICD-11) for mortality and morbidity statistics (version: 04/2019). Geneva: World Health Organization.

[ref85] Xu P. Xiong X. C. Xue Q. Tian Y. Peng Y. Zhang R.. (2014). Recognizing mild cognitive impairment based on network connectivity analysis of resting EEG with zero reference. Physiol. Meas. 35, 1279–1298. doi: 10.1088/0967-3334/35/7/1279, PMID: 24853724

[ref86] Yakovenko E. A. Rem A. V. Surushkina S. Y. Chutko L. S. (2021). Electroencephalographic signs of emotional burnout syndrome. Neurosci. Behav. Physiol. 51, 155–157. doi: 10.1007/s11055-021-01051-z

[ref87] Yue W. L. Ng K. K. Koh A. J. Perini F. Doshi K. Zhou J. H.. (2023). Mindfulness-based therapy improves brain functional network reconfiguration efficiency. Transl. Psychiatry 13:345. doi: 10.1038/s41398-023-02642-9, PMID: 37951943 PMC10640625

[ref88] Zhang D. Gao Z. Liang B. Li J. Cai Y. Wang Z.. (2019). Eyes closed elevates brain intrinsic activity of sensory dominance networks: a classifier discrimination analysis. Brain Connect. 9, 221–230. doi: 10.1089/brain.2018.0644, PMID: 30560680

[ref89] Zhang J. Hua Y. Xiu L. Oei T. P. Hu P. (2020). Resting state frontal alpha asymmetry predicts emotion regulation difficulties in impulse control. Personal. Individ. Differ. 159:109870. doi: 10.1016/j.paid.2020.109870

