# Supplementary material for: Functional connectivity in burnout syndrome: a resting-state EEG study
Source: Front Hum Neurosci. 2025 Feb 3;19:1481760. doi: 10.3389/fnhum.2025.1481760 (PMC11831065; doi:10.3389/fnhum.2025.1481760)
Supplement: Supplementary file 1 [file Table_1.DOCX]

**Table S1A.** Statistics for significant results in functional connectivity coherence in the eyes-open condition in alpha-3 sub-band (11-13 Hz) at the threshold of 0.5.

| **Numbers of electrodes** | ***U* statistic** | **Initial  *U* test  *p*-value** | **Corrected *U* test *p*-value** | **Median Control group** | **Median Burnout group** | **Difference  in coherence** |
| --- | --- | --- | --- | --- | --- | --- |
| E2_E5 | 719.0 | <0.001 | 0.048 | 0.721 | 0.557 | -0.163 |
| E2_E6 | 709.0 | <0.001 | 0.047 | 0.610 | 0.445 | -0.165 |
| E2_E13 | 717.0 | <0.001 | 0.047 | 0.731 | 0.604 | -0.127 |
| E2_E15 | 703.0 | <0.001 | 0.047 | 0.521 | 0.387 | -0.134 |
| E2_E206 | 718.0 | <0.001 | 0.048 | 0.607 | 0.453 | -0.155 |
| E2_E207 | 722.0 | <0.001 | 0.048 | 0.506 | 0.379 | -0.127 |
| E2_E214 | 730.0 | <0.001 | 0.048 | 0.733 | 0.638 | -0.095 |
| E2_E215 | 637.0 | <0.001 | 0.038 | 0.688 | 0.533 | -0.155 |
| E3_E5 | 704.0 | <0.001 | 0.047 | 0.911 | 0.858 | -0.054 |
| E3_E6 | 722.0 | <0.001 | 0.048 | 0.831 | 0.740 | -0.091 |
| E3_E206 | 739.0 | 0.001 | 0.048 | 0.763 | 0.667 | -0.097 |
| E3_E207 | 688.0 | <0.001 | 0.043 | 0.750 | 0.665 | -0.085 |
| E3_E214 | 652.0 | <0.001 | 0.042 | 0.885 | 0.807 | -0.078 |
| E3_E215 | 577.0 | <0.001 | 0.036 | 0.876 | 0.767 | -0.110 |
| E3_E223 | 710.0 | <0.001 | 0.047 | 0.972 | 0.941 | -0.031 |
| E3_E224 | 657.0 | <0.001 | 0.042 | 0.942 | 0.888 | -0.054 |
| E4_E5 | 697.0 | <0.001 | 0.044 | 0.987 | 0.971 | -0.016 |
| E4_E18 | 736.0 | <0.001 | 0.048 | 0.847 | 0.759 | -0.088 |
| E4_E31 | 717.0 | <0.001 | 0.047 | 0.648 | 0.431 | -0.217 |
| E4_E214 | 725.0 | <0.001 | 0.048 | 0.910 | 0.850 | -0.061 |
| E4_E215 | 611.0 | <0.001 | 0.036 | 0.949 | 0.913 | -0.036 |
| E4_E223 | 725.0 | <0.001 | 0.048 | 0.963 | 0.932 | -0.030 |
| E4_E224 | 602.0 | <0.001 | 0.036 | 0.985 | 0.965 | -0.020 |
| E5_E10 | 739.0 | 0.001 | 0.048 | 0.644 | 0.526 | -0.117 |
| E5_E11 | 682.0 | <0.001 | 0.043 | 0.862 | 0.771 | -0.090 |
| E5_E18 | 672.0 | <0.001 | 0.043 | 0.819 | 0.686 | -0.133 |
| E5_E25 | 716.0 | <0.001 | 0.047 | 0.803 | 0.650 | -0.152 |
| E5_E31 | 651.0 | <0.001 | 0.042 | 0.621 | 0.372 | -0.249 |
| E5_E32 | 714.0 | <0.001 | 0.047 | 0.709 | 0.580 | -0.129 |
| E5_E222 | 712.0 | <0.001 | 0.047 | 0.779 | 0.645 | -0.134 |
| E6_E10 | 722.0 | <0.001 | 0.048 | 0.567 | 0.409 | -0.159 |
| E6_E11 | 676.0 | <0.001 | 0.043 | 0.793 | 0.636 | -0.157 |
| E6_E18 | 664.0 | <0.001 | 0.042 | 0.748 | 0.585 | -0.163 |
| E6_E19 | 697.0 | <0.001 | 0.044 | 0.880 | 0.770 | -0.110 |
| E6_E25 | 704.0 | <0.001 | 0.047 | 0.754 | 0.579 | -0.175 |
| E6_E26 | 728.0 | <0.001 | 0.048 | 0.806 | 0.728 | -0.079 |
| E6_E31 | 633.0 | <0.001 | 0.038 | 0.545 | 0.330 | -0.214 |
| E6_E32 | 705.0 | <0.001 | 0.047 | 0.707 | 0.519 | -0.188 |
| E6_E37 | 707.0 | <0.001 | 0.047 | 0.579 | 0.419 | -0.160 |
| E6_E222 | 737.0 | 0.001 | 0.048 | 0.676 | 0.519 | -0.156 |
| E7_E11 | 726.0 | <0.001 | 0.048 | 0.655 | 0.535 | -0.120 |
| E7_E18 | 693.0 | <0.001 | 0.044 | 0.627 | 0.426 | -0.201 |
| E7_E19 | 738.0 | 0.001 | 0.048 | 0.758 | 0.638 | -0.120 |
| E7_E25 | 732.0 | <0.001 | 0.048 | 0.649 | 0.447 | -0.202 |
| E7_E26 | 739.0 | 0.001 | 0.048 | 0.751 | 0.622 | -0.129 |
| E7_E31 | 706.0 | <0.001 | 0.047 | 0.505 | 0.236 | -0.269 |
| E7_E32 | 731.0 | <0.001 | 0.048 | 0.647 | 0.411 | -0.237 |
| E7_E37 | 707.0 | <0.001 | 0.047 | 0.548 | 0.333 | -0.214 |
| E8_E19 | 742.0 | 0.001 | 0.049 | 0.598 | 0.443 | -0.155 |
| E8_E26 | 735.0 | <0.001 | 0.048 | 0.592 | 0.430 | -0.162 |
| E10_E206 | 679.0 | <0.001 | 0.043 | 0.507 | 0.337 | -0.170 |
| E10_E214 | 707.0 | <0.001 | 0.047 | 0.643 | 0.478 | -0.165 |
| E10_E215 | 663.0 | <0.001 | 0.042 | 0.615 | 0.418 | -0.197 |
| E11_E12 | 733.0 | <0.001 | 0.048 | 0.950 | 0.916 | -0.034 |
| E11_E13 | 719.0 | <0.001 | 0.048 | 0.879 | 0.812 | -0.067 |
| E11_E15 | 697.0 | <0.001 | 0.044 | 0.699 | 0.582 | -0.117 |
| E11_E207 | 698.0 | <0.001 | 0.044 | 0.656 | 0.537 | -0.119 |
| E11_E214 | 698.0 | <0.001 | 0.044 | 0.788 | 0.670 | -0.118 |
| E11_E215 | 665.0 | <0.001 | 0.042 | 0.783 | 0.674 | -0.108 |
| E12_E215 | 706.0 | <0.001 | 0.047 | 0.897 | 0.826 | -0.071 |
| E13_E18 | 720.0 | <0.001 | 0.048 | 0.836 | 0.756 | -0.080 |
| E13_E19 | 726.0 | <0.001 | 0.048 | 0.939 | 0.904 | -0.035 |
| E13_E25 | 739.0 | 0.001 | 0.048 | 0.845 | 0.722 | -0.123 |
| E13_E31 | 669.0 | <0.001 | 0.043 | 0.690 | 0.460 | -0.229 |
| E13_E32 | 734.0 | <0.001 | 0.048 | 0.792 | 0.691 | -0.102 |
| E13_E222 | 713.0 | <0.001 | 0.047 | 0.723 | 0.607 | -0.116 |
| E14_E18 | 742.0 | 0.001 | 0.049 | 0.775 | 0.640 | -0.135 |
| E14_E25 | 729.0 | <0.001 | 0.048 | 0.800 | 0.689 | -0.111 |
| E14_E31 | 650.0 | <0.001 | 0.042 | 0.650 | 0.384 | -0.266 |
| E14_E32 | 727.0 | <0.001 | 0.048 | 0.769 | 0.624 | -0.144 |
| E14_E37 | 719.0 | <0.001 | 0.048 | 0.695 | 0.540 | -0.155 |
| E15_E18 | 692.0 | <0.001 | 0.044 | 0.697 | 0.492 | -0.205 |
| E15_E19 | 697.0 | <0.001 | 0.044 | 0.848 | 0.733 | -0.115 |
| E15_E25 | 725.0 | <0.001 | 0.048 | 0.763 | 0.576 | -0.187 |
| E15_E26 | 655.0 | <0.001 | 0.042 | 0.840 | 0.720 | -0.120 |
| E15_E31 | 679.0 | <0.001 | 0.043 | 0.590 | 0.335 | -0.255 |
| E15_E32 | 733.0 | <0.001 | 0.048 | 0.746 | 0.574 | -0.173 |
| E15_E37 | 713.0 | <0.001 | 0.047 | 0.668 | 0.476 | -0.192 |
| E15_E222 | 740.0 | 0.001 | 0.048 | 0.535 | 0.374 | -0.160 |
| E16_E18 | 720.0 | <0.001 | 0.048 | 0.559 | 0.402 | -0.157 |
| E16_E19 | 714.0 | <0.001 | 0.047 | 0.723 | 0.605 | -0.118 |
| E16_E25 | 739.0 | 0.001 | 0.048 | 0.647 | 0.478 | -0.169 |
| E16_E26 | 705.0 | <0.001 | 0.047 | 0.736 | 0.645 | -0.091 |
| E16_E31 | 713.0 | <0.001 | 0.047 | 0.535 | 0.231 | -0.304 |
| E18_E114 | 721.0 | <0.001 | 0.048 | 0.500 | 0.329 | -0.172 |
| E18_E115 | 623.0 | <0.001 | 0.038 | 0.600 | 0.421 | -0.179 |
| E18_E123 | 628.0 | <0.001 | 0.038 | 0.576 | 0.383 | -0.193 |
| E18_E124 | 704.0 | <0.001 | 0.047 | 0.634 | 0.468 | -0.166 |
| E18_E135 | 714.0 | <0.001 | 0.047 | 0.534 | 0.317 | -0.217 |
| E18_E136 | 643.0 | <0.001 | 0.039 | 0.598 | 0.418 | -0.181 |
| E18_E137 | 730.0 | <0.001 | 0.048 | 0.628 | 0.470 | -0.158 |
| E18_E147 | 685.0 | <0.001 | 0.043 | 0.531 | 0.313 | -0.218 |
| E18_E148 | 685.0 | <0.001 | 0.043 | 0.578 | 0.396 | -0.182 |
| E18_E206 | 695.0 | <0.001 | 0.044 | 0.627 | 0.427 | -0.199 |
| E18_E207 | 644.0 | <0.001 | 0.039 | 0.601 | 0.423 | -0.178 |
| E18_E213 | 740.0 | 0.001 | 0.048 | 0.705 | 0.577 | -0.129 |
| E18_E214 | 643.0 | <0.001 | 0.039 | 0.702 | 0.575 | -0.127 |
| E18_E215 | 605.0 | <0.001 | 0.036 | 0.755 | 0.552 | -0.203 |
| E18_E222 | 702.0 | <0.001 | 0.047 | 0.808 | 0.725 | -0.082 |
| E18_E223 | 611.0 | <0.001 | 0.036 | 0.842 | 0.724 | -0.118 |
| E18_E224 | 662.0 | <0.001 | 0.042 | 0.832 | 0.695 | -0.137 |
| E19_E207 | 728.0 | <0.001 | 0.048 | 0.715 | 0.604 | -0.112 |
| E19_E215 | 682.0 | <0.001 | 0.043 | 0.839 | 0.718 | -0.120 |
| E20_E31 | 678.0 | <0.001 | 0.043 | 0.780 | 0.612 | -0.168 |
| E21_E31 | 725.0 | <0.001 | 0.048 | 0.730 | 0.515 | -0.216 |
| E22_E26 | 728.0 | <0.001 | 0.048 | 0.912 | 0.833 | -0.079 |
| E22_E31 | 672.0 | <0.001 | 0.043 | 0.662 | 0.385 | -0.277 |
| E23_E31 | 694.0 | <0.001 | 0.044 | 0.591 | 0.290 | -0.301 |
| E25_E123 | 724.0 | <0.001 | 0.048 | 0.522 | 0.399 | -0.123 |
| E25_E136 | 720.0 | <0.001 | 0.048 | 0.581 | 0.442 | -0.139 |
| E25_E147 | 722.0 | <0.001 | 0.048 | 0.536 | 0.342 | -0.194 |
| E25_E148 | 723.0 | <0.001 | 0.048 | 0.583 | 0.439 | -0.143 |
| E25_E207 | 714.0 | <0.001 | 0.047 | 0.583 | 0.423 | -0.160 |
| E25_E214 | 696.0 | <0.001 | 0.044 | 0.614 | 0.448 | -0.166 |
| E25_E215 | 677.0 | <0.001 | 0.043 | 0.698 | 0.517 | -0.182 |
| E25_E224 | 729.0 | <0.001 | 0.048 | 0.765 | 0.613 | -0.152 |
| E26_E31 | 682.0 | <0.001 | 0.043 | 0.873 | 0.779 | -0.094 |
| E26_E32 | 689.0 | <0.001 | 0.043 | 0.969 | 0.934 | -0.035 |
| E26_E207 | 732.0 | <0.001 | 0.048 | 0.633 | 0.513 | -0.120 |
| E26_E215 | 713.0 | <0.001 | 0.047 | 0.736 | 0.615 | -0.120 |
| E28_E31 | 678.0 | <0.001 | 0.043 | 0.717 | 0.506 | -0.211 |
| E29_E31 | 675.0 | <0.001 | 0.043 | 0.660 | 0.391 | -0.268 |
| E29_E39 | 733.0 | <0.001 | 0.048 | 0.900 | 0.830 | -0.070 |
| E29_E55 | 738.0 | 0.001 | 0.048 | 0.511 | 0.385 | -0.126 |
| E29_E151 | 721.0 | <0.001 | 0.048 | 0.637 | 0.517 | -0.121 |
| E29_E152 | 701.0 | <0.001 | 0.046 | 0.577 | 0.461 | -0.116 |
| E30_E31 | 716.0 | <0.001 | 0.047 | 0.534 | 0.288 | -0.245 |
| E30_E55 | 713.0 | <0.001 | 0.047 | 0.544 | 0.364 | -0.180 |
| E31_E32 | 625.0 | <0.001 | 0.038 | 0.943 | 0.872 | -0.071 |
| E31_E116 | 680.0 | <0.001 | 0.043 | 0.512 | 0.351 | -0.161 |
| E31_E125 | 705.0 | <0.001 | 0.047 | 0.592 | 0.412 | -0.180 |
| E31_E136 | 578.0 | <0.001 | 0.036 | 0.520 | 0.282 | -0.238 |
| E31_E137 | 605.0 | <0.001 | 0.036 | 0.575 | 0.337 | -0.238 |
| E31_E138 | 645.0 | <0.001 | 0.039 | 0.637 | 0.389 | -0.248 |
| E31_E148 | 572.0 | <0.001 | 0.036 | 0.525 | 0.298 | -0.227 |
| E31_E149 | 623.0 | <0.001 | 0.038 | 0.542 | 0.345 | -0.197 |
| E31_E150 | 696.0 | <0.001 | 0.044 | 0.556 | 0.395 | -0.161 |
| E31_E215 | 632.0 | <0.001 | 0.038 | 0.536 | 0.279 | -0.256 |
| E31_E223 | 719.0 | <0.001 | 0.048 | 0.564 | 0.385 | -0.179 |
| E31_E224 | 664.0 | <0.001 | 0.042 | 0.567 | 0.378 | -0.189 |
| E32_E136 | 716.0 | <0.001 | 0.047 | 0.584 | 0.454 | -0.129 |
| E32_E147 | 719.0 | <0.001 | 0.048 | 0.534 | 0.357 | -0.177 |
| E32_E148 | 686.0 | <0.001 | 0.043 | 0.629 | 0.457 | -0.172 |
| E32_E149 | 734.0 | <0.001 | 0.048 | 0.628 | 0.477 | -0.151 |
| E32_E158 | 682.0 | <0.001 | 0.043 | 0.532 | 0.389 | -0.143 |
| E32_E159 | 681.0 | <0.001 | 0.043 | 0.548 | 0.384 | -0.164 |
| E32_E207 | 693.0 | <0.001 | 0.044 | 0.518 | 0.331 | -0.187 |
| E32_E215 | 660.0 | <0.001 | 0.042 | 0.612 | 0.425 | -0.187 |
| E32_E224 | 699.0 | <0.001 | 0.045 | 0.650 | 0.503 | -0.147 |
| E34_E54 | 726.0 | <0.001 | 0.048 | 0.532 | 0.395 | -0.137 |
| E35_E54 | 714.0 | <0.001 | 0.047 | 0.529 | 0.399 | -0.129 |
| E37_E148 | 726.0 | <0.001 | 0.048 | 0.581 | 0.439 | -0.142 |
| E37_E149 | 721.0 | <0.001 | 0.048 | 0.590 | 0.473 | -0.117 |
| E37_E158 | 697.0 | <0.001 | 0.044 | 0.551 | 0.395 | -0.156 |
| E37_E159 | 677.0 | <0.001 | 0.043 | 0.558 | 0.418 | -0.140 |
| E42_E49 | 731.0 | <0.001 | 0.048 | 0.862 | 0.782 | -0.080 |
| E42_E151 | 728.0 | <0.001 | 0.048 | 0.602 | 0.468 | -0.134 |
| E46_E149 | 739.0 | 0.001 | 0.048 | 0.511 | 0.379 | -0.132 |
| E46_E150 | 737.0 | 0.001 | 0.048 | 0.588 | 0.469 | -0.120 |
| E46_E160 | 727.0 | <0.001 | 0.048 | 0.533 | 0.422 | -0.111 |
| E54_E139 | 716.0 | <0.001 | 0.047 | 0.568 | 0.438 | -0.130 |
| E54_E140 | 728.0 | <0.001 | 0.048 | 0.637 | 0.495 | -0.142 |
| E76_E77 | 661.0 | <0.001 | 0.042 | 0.951 | 0.925 | -0.026 |
| E85_E87 | 717.0 | <0.001 | 0.047 | 0.786 | 0.710 | -0.077 |
| E85_E99 | 739.0 | 0.001 | 0.048 | 0.639 | 0.540 | -0.099 |
| E93_E164 | 734.0 | <0.001 | 0.048 | 0.370 | 0.520 | 0.150 |
| E96_E98 | 727.0 | <0.001 | 0.048 | 0.786 | 0.679 | -0.107 |
| E101_E138 | 735.0 | <0.001 | 0.048 | 0.592 | 0.454 | -0.138 |
| E108_E115 | 733.0 | <0.001 | 0.048 | 0.881 | 0.822 | -0.059 |
| E128_E138 | 728.0 | <0.001 | 0.048 | 0.783 | 0.713 | -0.069 |
| E149_E160 | 742.0 | 0.001 | 0.049 | 0.839 | 0.782 | -0.057 |
| E215_E221 | 732.0 | <0.001 | 0.048 | 0.560 | 0.351 | -0.209 |
| E215_E222 | 637.0 | <0.001 | 0.038 | 0.753 | 0.622 | -0.131 |
| E215_E223 | 741.0 | 0.001 | 0.049 | 0.922 | 0.881 | -0.041 |
| E222_E224 | 726.0 | <0.001 | 0.048 | 0.853 | 0.752 | -0.101 |

*Note:* E – electrode; *U* statistic for Mann-Whitney test; corrected *p*-value for *U* test for multiple comparisons using the Benjamini-Hochberg false discovery rate (FDR) method; Difference in coherence calculated as the difference between the median of the burnout group and the control group.
