# Supplementary material for: Functional connectivity in burnout syndrome: a resting-state EEG study
Source: Front Hum Neurosci. 2025 Feb 3;19:1481760. doi: 10.3389/fnhum.2025.1481760 (PMC11831065; doi:10.3389/fnhum.2025.1481760)
Supplement: Supplementary file 2 [file Table_2.DOCX]

**Table S1B.** Statistics for significant results in functional connectivity coherence in the eyes-open condition in alpha-3 sub-band (11-13 Hz) at the threshold of 0.7.

| **Numbers of electrodes** | ***U* statistic** | **Initial  *U* test  *p*-value** | **Corrected** ***U* test *p*-value** | **Median Control group** | **Median Burnout group** | **Difference  in coherence** |
| --- | --- | --- | --- | --- | --- | --- |
| E2_E5 | 719.0 | <0.001 | 0.048 | 0.721 | 0.557 | -0.163 |
| E2_E13 | 717.0 | <0.001 | 0.047 | 0.731 | 0.604 | -0.127 |
| E2_E214 | 730.0 | <0.001 | 0.048 | 0.733 | 0.638 | -0.095 |
| E3_E5 | 704.0 | <0.001 | 0.047 | 0.911 | 0.858 | -0.054 |
| E3_E6 | 722.0 | <0.001 | 0.048 | 0.831 | 0.740 | -0.091 |
| E3_E206 | 739.0 | 0.001 | 0.048 | 0.763 | 0.667 | -0.097 |
| E3_E207 | 688.0 | <0.001 | 0.043 | 0.750 | 0.665 | -0.085 |
| E3_E214 | 652.0 | <0.001 | 0.042 | 0.885 | 0.807 | -0.078 |
| E3_E215 | 577.0 | <0.001 | 0.036 | 0.876 | 0.767 | -0.110 |
| E3_E223 | 710.0 | <0.001 | 0.047 | 0.972 | 0.941 | -0.031 |
| E3_E224 | 657.0 | <0.001 | 0.042 | 0.942 | 0.888 | -0.054 |
| E4_E5 | 697.0 | <0.001 | 0.044 | 0.987 | 0.971 | -0.016 |
| E4_E18 | 736.0 | <0.001 | 0.048 | 0.847 | 0.759 | -0.088 |
| E4_E214 | 725.0 | <0.001 | 0.048 | 0.910 | 0.850 | -0.061 |
| E4_E215 | 611.0 | <0.001 | 0.036 | 0.949 | 0.913 | -0.036 |
| E4_E223 | 725.0 | <0.001 | 0.048 | 0.963 | 0.932 | -0.030 |
| E4_E224 | 602.0 | <0.001 | 0.036 | 0.985 | 0.965 | -0.020 |
| E5_E11 | 682.0 | <0.001 | 0.043 | 0.862 | 0.771 | -0.090 |
| E5_E18 | 672.0 | <0.001 | 0.043 | 0.819 | 0.686 | -0.133 |
| E5_E25 | 716.0 | <0.001 | 0.047 | 0.803 | 0.650 | -0.152 |
| E5_E32 | 714.0 | <0.001 | 0.047 | 0.709 | 0.580 | -0.129 |
| E5_E222 | 712.0 | <0.001 | 0.047 | 0.779 | 0.645 | -0.134 |
| E6_E11 | 676.0 | <0.001 | 0.043 | 0.793 | 0.636 | -0.157 |
| E6_E18 | 664.0 | <0.001 | 0.042 | 0.748 | 0.585 | -0.163 |
| E6_E19 | 697.0 | <0.001 | 0.044 | 0.880 | 0.770 | -0.110 |
| E6_E25 | 704.0 | <0.001 | 0.047 | 0.754 | 0.579 | -0.175 |
| E6_E26 | 728.0 | <0.001 | 0.048 | 0.806 | 0.728 | -0.079 |
| E6_E32 | 705.0 | <0.001 | 0.047 | 0.707 | 0.519 | -0.188 |
| E7_E19 | 738.0 | 0.001 | 0.048 | 0.758 | 0.638 | -0.120 |
| E7_E26 | 739.0 | 0.001 | 0.048 | 0.751 | 0.622 | -0.129 |
| E11_E12 | 733.0 | <0.001 | 0.048 | 0.950 | 0.916 | -0.034 |
| E11_E13 | 719.0 | <0.001 | 0.048 | 0.879 | 0.812 | -0.067 |
| E11_E214 | 698.0 | <0.001 | 0.044 | 0.788 | 0.670 | -0.118 |
| E11_E215 | 665.0 | <0.001 | 0.042 | 0.783 | 0.674 | -0.108 |
| E12_E215 | 706.0 | <0.001 | 0.047 | 0.897 | 0.826 | -0.071 |
| E13_E18 | 720.0 | <0.001 | 0.048 | 0.836 | 0.756 | -0.080 |
| E13_E19 | 726.0 | <0.001 | 0.048 | 0.939 | 0.904 | -0.035 |
| E13_E25 | 739.0 | 0.001 | 0.048 | 0.845 | 0.722 | -0.123 |
| E13_E32 | 734.0 | <0.001 | 0.048 | 0.792 | 0.691 | -0.102 |
| E13_E222 | 713.0 | <0.001 | 0.047 | 0.723 | 0.607 | -0.116 |
| E14_E18 | 742.0 | 0.001 | 0.049 | 0.775 | 0.640 | -0.135 |
| E14_E25 | 729.0 | <0.001 | 0.048 | 0.800 | 0.689 | -0.111 |
| E14_E32 | 727.0 | <0.001 | 0.048 | 0.769 | 0.624 | -0.144 |
| E15_E19 | 697.0 | <0.001 | 0.044 | 0.848 | 0.733 | -0.115 |
| E15_E25 | 725.0 | <0.001 | 0.048 | 0.763 | 0.576 | -0.187 |
| E15_E32 | 733.0 | <0.001 | 0.048 | 0.746 | 0.574 | -0.173 |
| E16_E19 | 714.0 | <0.001 | 0.047 | 0.723 | 0.605 | -0.118 |
| E16_E26 | 705.0 | <0.001 | 0.047 | 0.736 | 0.645 | -0.091 |
| E18_E213 | 740.0 | 0.001 | 0.048 | 0.705 | 0.577 | -0.129 |
| E18_E214 | 643.0 | <0.001 | 0.039 | 0.702 | 0.575 | -0.127 |
| E18_E215 | 605.0 | <0.001 | 0.036 | 0.755 | 0.552 | -0.203 |
| E18_E222 | 702.0 | <0.001 | 0.047 | 0.808 | 0.725 | -0.082 |
| E18_E223 | 611.0 | <0.001 | 0.036 | 0.842 | 0.724 | -0.118 |
| E18_E224 | 662.0 | <0.001 | 0.042 | 0.832 | 0.695 | -0.137 |
| E19_E207 | 728.0 | <0.001 | 0.048 | 0.715 | 0.604 | -0.112 |
| E19_E215 | 682.0 | <0.001 | 0.043 | 0.839 | 0.718 | -0.120 |
| E20_E31 | 678.0 | <0.001 | 0.043 | 0.780 | 0.612 | -0.168 |
| E21_E31 | 725.0 | <0.001 | 0.048 | 0.730 | 0.515 | -0.216 |
| E22_E26 | 728.0 | <0.001 | 0.048 | 0.912 | 0.833 | -0.079 |
| E25_E224 | 729.0 | <0.001 | 0.048 | 0.765 | 0.613 | -0.152 |
| E26_E31 | 682.0 | <0.001 | 0.043 | 0.873 | 0.779 | -0.094 |
| E26_E32 | 689.0 | <0.001 | 0.043 | 0.969 | 0.934 | -0.035 |
| E26_E215 | 713.0 | <0.001 | 0.047 | 0.736 | 0.615 | -0.120 |
| E28_E31 | 678.0 | <0.001 | 0.043 | 0.717 | 0.506 | -0.211 |
| E29_E39 | 733.0 | <0.001 | 0.048 | 0.900 | 0.830 | -0.070 |
| E31_E32 | 625.0 | <0.001 | 0.038 | 0.943 | 0.872 | -0.071 |
| E42_E49 | 731.0 | <0.001 | 0.048 | 0.862 | 0.782 | -0.080 |
| E76_E77 | 661.0 | <0.001 | 0.042 | 0.951 | 0.925 | -0.026 |
| E85_E87 | 717.0 | <0.001 | 0.047 | 0.786 | 0.710 | -0.077 |
| E96_E98 | 727.0 | <0.001 | 0.048 | 0.786 | 0.679 | -0.107 |
| E108_E115 | 733.0 | <0.001 | 0.048 | 0.881 | 0.822 | -0.059 |
| E128_E138 | 728.0 | <0.001 | 0.048 | 0.783 | 0.713 | -0.069 |
| E149_E160 | 742.0 | 0.001 | 0.049 | 0.839 | 0.782 | -0.057 |
| E215_E222 | 637.0 | <0.001 | 0.038 | 0.753 | 0.622 | -0.131 |
| E215_E223 | 741.0 | 0.001 | 0.049 | 0.922 | 0.881 | -0.041 |
| E222_E224 | 726.0 | <0.001 | 0.048 | 0.853 | 0.752 | -0.101 |

*Note:* E – electrode; *U* statistic for Mann-Whitney test; corrected *p*-value for *U* test for multiple comparisons using the Benjamini-Hochberg false discovery rate (FDR) method; Difference in coherence calculated as the difference between the median of the burnout group and the control group.
